# Bilateral Ureteral Obstruction Causing Acute Kidney Injury and Resultant Metformin Toxicity

**DOI:** 10.7759/cureus.19635

**Published:** 2021-11-16

**Authors:** Umar Rashid, Erin M Marra, Vu H Tran

**Affiliations:** 1 Emergency Medicine, Graduate Medical Education, Aventura Hospital and Medical Center, Aventura, USA

**Keywords:** emergency medicine, metformin, high anion gap metabolic acidosis, ureteral calculi, aki

## Abstract

A 55-year-old male with a past medical history of type 2 diabetes mellitus on metformin presented to the emergency department (ED) due to shortness of breath and three days of lumbar back pain. Workup revealed bilateral obstructing ureteral stones causing bilateral hydronephrosis, acute kidney injury (AKI), and profound anion gap metabolic acidosis due to concomitant metformin-associated lactic acidosis (MALA). In the ED, the patient developed profound shock refractory to fluid resuscitation, requiring initiation of multiple vasopressors, and stress dose steroids. He was transferred to the interventional radiology suite for bilateral percutaneous nephrostomy tubes and only improved once continuous renal replacement therapy was initiated.

## Introduction

Nephrolithiasis is a disease frequently treated in the emergency department due to the pain associated with the condition. There are an estimated 1.2 million annual visits per year in the US alone [1]. In a lifetime it is expected that one in 10 people will suffer from nephrolithiasis [2]. It commonly presents with severe flank and lower back pain radiating to the groin. Additional symptoms may include hematuria, nausea, and vomiting. Small stones generally pass without intervention and are treated with observation and pain management, whereas larger stones tend to cause complications when obstruction occurs. Such complications can cause acute kidney injury (AKI) when both urinary outflow tracts are blocked or when one tract is disrupted in a single functional kidney [3]. In normal instances, unilateral obstruction does not cause AKI due to compensation from the unaffected kidney. However, in our case, we had the unusual presentation of bilateral ureteric obstruction from stones causing bilateral hydronephrosis. According to our literature search, there have been few such incidents reported.

## Case presentation

We describe the case of a 55-year-old male who presented to the emergency department via emergency medical services for the chief complaint of sudden onset shortness of breath that woke him from his sleep just prior to arrival. He reported three days of non-radiating lumbar back pain and two episodes of non-bloody emesis leading up to this event. His medical history included hypertension and type 2 diabetes mellitus. His current medications were metformin, amlodipine, losartan, and atenolol. Initial vital signs revealed heart rate (HR) 75, respiratory rate (RR) 29, blood pressure (BP) 119/62, and oxygen saturation 99% on 2L nasal cannula. Temperature was 36.3°C.

Physical examination was significant for an ill-appearing male patient who was anxious and tachypneic. He also had significant work of breathing with retractions and abdominal breathing. Lungs were clear to auscultation, with no wheezing, rhonchi, or rales. Abdominal exam revealed mild epigastric tenderness with no rebound, guarding, or palpable pulsatile mass. No costovertebral angle (CVA) tenderness or midline tenderness was elicited. Neurological exam revealed no focal deficits.

Due to his presentation and multiple comorbidities we had significant concern for the possible acute coronary syndrome, dissection, sepsis, or pulmonary embolism. Blood work including complete blood count (CBC), comprehensive metabolic panel (CMP), lactic acid, troponin, urine analysis (UA) was ordered. CT-angiogram of his chest, abdomen, and pelvis was also ordered.

**Figure 1:**
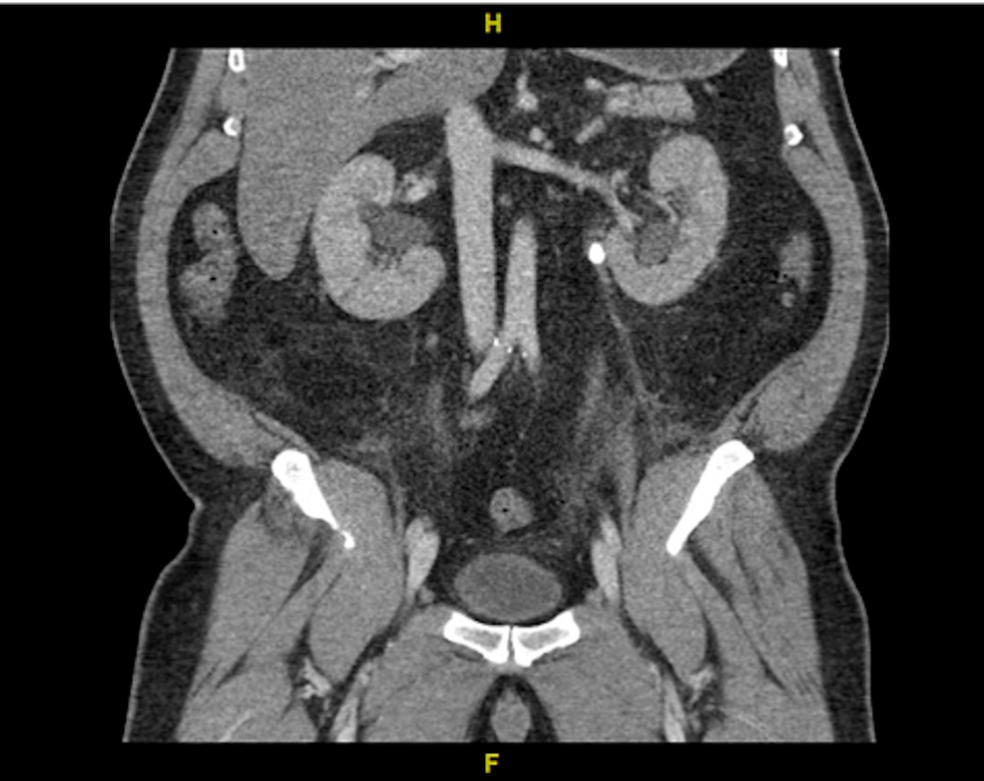
Coronal view: Left obstructing ureteropelvic junction calculi and bilateral hydronephrosis

**Figure 2:**
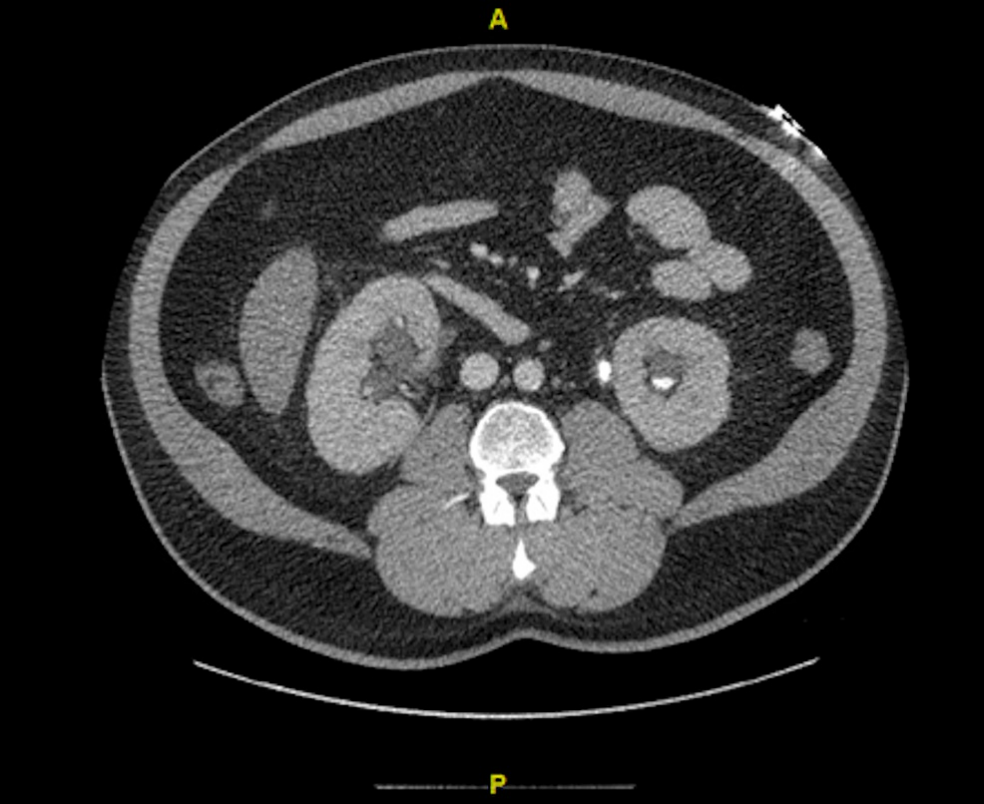
Transverse view: Left ureteropelvic junction calculi

**Figure 3:**
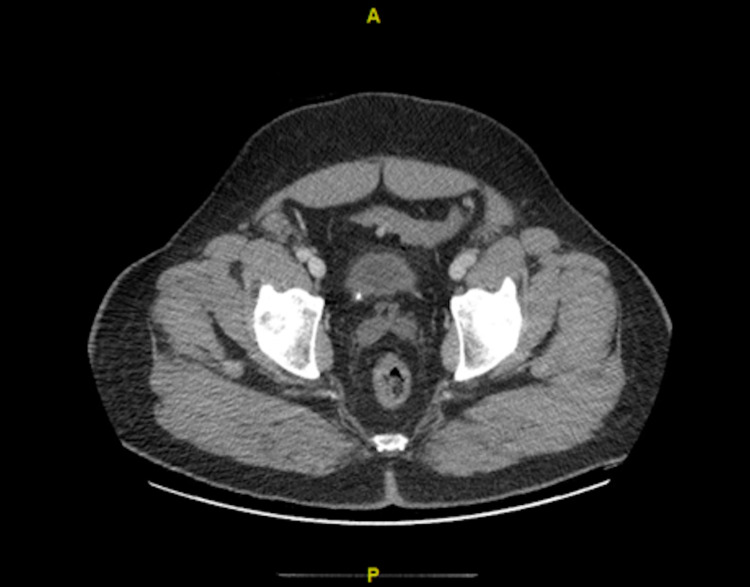
Transverse view: Right ureterovesical junction stone

Complete blood count revealed a white blood cell count of 20.4 x 10^3^/uL, hemoglobin of 11.2 g/dL, and platelet count of 376 x 10^3^/uL. Comprehensive metabolic panel was significant for a sodium of 145 mmol/L, potassium 6.1 mmol/L, chloride 100 mmol/l, bicarbonate <7 mmol, blood urea nitrogen (BUN) 67 mg/dl, creatinine 14.7 mg/dL, calcium 9.1 mg/dL, and glucose of 165. Troponin I was <0.012 ng/mL. Initial lactic acid was recorded at 13.9 mmol/L. An arterial blood gas revealed severe anion gap metabolic acidosis with a pH of 6.9, pCO2 of 22.3 mmHg, pO2 of 105.5 mmHg, HCO3 of 5.3 meq/L, and a Base Excess of -24.8 mmol/l. The patient was anuric at this time. Initial EKG showed normal sinus rhythm (NSR), nonspecific ST abnormalities, vent rate 73, QTC 471. The CT scan revealed an obstructing stone at the left proximal ureteropelvic junction (Figures 1, 2) and at the right ureterovesical junction (Figure 3), with resultant bilateral hydronephrosis (Figure 1).

While in the ED the patient progressively became hypotensive and was given broad-spectrum antibiotics, Vancomycin and Cefepime, due to concern for sepsis and aggressive fluid resuscitation was initiated. The patient did not respond to fluid resuscitation and was started initially on Levophed. In addition, the patient received 100 mL of 8.4% sodium bicarbonate and 2 grams of calcium gluconate for severe metabolic acidosis and hyperkalemia. Due to the expected clinical decline, the patient was intubated in the emergency department. Critical Care, Nephrology, and Urology were consulted emergently in the ED. After initial stabilization, the patient was taken for immediate bilateral percutaneous nephrostomy (PCN) placement by Interventional Radiology (IR) and placement of a trialysis catheter for initiation of hemodialysis afterward. Urinalysis from the PCN revealed hematuria, but no evidence of infection.

After successful placement of bilateral PCN, the patient was transferred to the intensive care unit (ICU) in critical condition. He was maintained on multiple vasopressors consisting of combinations of norepinephrine, vasopressin, epinephrine, and phenylephrine along with stress dose hydrocortisone. Repeat blood gas and labs revealed a worsening metabolic and lactic acidosis with a repeat pH of 6.7 and a lactic acid of 15.9. The patient was started on hemodialysis with a high bicarbonate dialysate for emergent clearance of hyperkalemia and refractory acidosis. After hemodialysis, the patient’s pH was 7.1, despite a worsening lactic acidosis of 17.0 mmol/L. Overnight the patient was converted to continuous venous-venous hemodialysis, a form of continuous renal replacement therapy (CRRT). Within 24 hours the patient’s acidemia corrected and blood pressure stabilized allowing for a de-escalation of vasopressors. A repeat chemistry revealed recovering renal function along with urine production in the nephrostomy tubes. On hospital day 3, the patient was successfully extubated, weaned off all vasopressors, and CRRT was stopped. Blood and urine cultures taken prior to dialysis were negative after five days. On hospital day 6, Urology performed a cystoscopy with bilateral ureteral stent placement and removal of bilateral percutaneous nephrostomy tubes. On hospital day 8, the patient had made a remarkable recovery from being critically ill. His creatinine was 1.5, BUN 27, and he was discharged home with urology follow-up in 1-2 weeks.

## Discussion

Immediate intervention to adequately resuscitate the patient and decompress the kidney is needed to avoid permanent renal damage. The two methods commonly used are percutaneous nephrostomy tube placement or retrograde ureteral stent insertion [4]. The optimal strategy remains controversial [5]. In our case, the urologist recommended immediate bilateral percutaneous nephrostomy tube placement by IR due to our patient being hemodynamically unstable and with future plans of ureteral stent placement when stable.

Urosepsis was high on our differential given the patient’s presentation, however no source of infection was discovered, and all cultures taken in the emergency department remained negative. After literature search on bilateral ureteral obstruction secondary to ureterolithiasis causing bilateral hydronephrosis, we found that there were only a few reported cases but none causing a shocked state requiring max pressors such as our patient. All reported cases resolved with PCN placement, whereas our case had worsening lactic acidosis. With a thorough review of previous medical records and history from the patient, we believe drug toxicity might have played a role in this patient. The profound shock state with lactic acidosis we suspect was due to concurrent metformin toxicity resulting from the AKI caused by the ureteral obstruction. We did not have the capability to send a formal metformin level, however, we did know that the patient continued to take his metformin over the ensuing days while suffering from AKI.

Lactic acidosis due to metformin is a complex process and is due to the drug inhibiting mitochondrial cellular respiration, which in turn leads to an increase in anaerobic metabolism and lactate levels [6,7]. Metformin-associated lactic acidosis (MALA) is a rare but life-threatening event, defined as arterial pH of <7.35 and a lactate concentration of >5 mmol/l in the setting of acute or chronic metformin exposure [6]. Metformin is generally regarded as a safe drug with common adverse effects being diarrhea, nausea, vomiting, and abdominal bloating [6-9]. It is the most commonly prescribed agent for newly diagnosed type 2 diabetes with a potentially rare but life-threatening complication of lactic acidosis [10]. It has been previously reported that toxic concentrations of the drug can occur when there is either an acute overdose or from renal dysfunction preventing excretion, leading to increased metformin plasma levels [6,7]. Its toxicity is associated with a mortality rate of 30% [11]. Clinical findings may not always correlate to the extent of the metabolic derangement present and patients who appear stable may deteriorate rapidly [8]. Patients with metformin toxicity should receive hemodialysis if they present with any of the following: Lactate >20 mmol/L, pH ≤ 7.0, shock, failure of supportive measures, and decreased level of consciousness [11]. CRRT has been shown in recent case reports to lead to positive outcomes and seems to be the emerging treatment of choice [12-14].

## Conclusions

Drug toxicities secondary to acute kidney injury are important to consider in a patient who presents with profound acidosis. A vital part of the patient encounter is a thorough history and medication reconciliation. Our patient presented with rare bilateral ureteral obstruction causing the primary insult to the kidneys which we believe was compounded by metformin toxicity leading to profound shock. Although we were unable to send formal levels, due to the clinical presentation and lack of other sources of infection we believe metformin toxicity should be considered in this case. Other causes of sepsis were ruled out before coming to this conclusion. Our case shows that aggressive resuscitation and CRRT can lead to positive outcomes in critically ill patients with suspected MALA. Further research is needed but the few published case reports of people diagnosed with MALA who get CRRT show promising results.
